# Feasibility of Interstitial Fluid Ketone Monitoring with Microneedles

**DOI:** 10.3390/metabo12050424

**Published:** 2022-05-10

**Authors:** Robert M. Taylor, Justin T. Baca

**Affiliations:** Department of Emergency Medicine, The University of New Mexico, Albuquerque, NM 87131, USA; rmtaylor@salud.unm.edu

**Keywords:** interstitial fluid, ISF, ketone, ketosis, diabetic ketoacidosis, DKA, microneedle, dermal, wearable

## Abstract

Diabetic ketoacidosis (DKA) is one of the most dangerous and costly complications of diabetes, accounting for approximately 50% of deaths in diabetic individuals under 24 years. This results in over 130,000 hospital admissions yearly and costs the USA over USD 2.4 billion annually. Earlier diagnosis, treatment, and management of DKA are of critical importance to achieving better patient outcomes and preventing prolonged hospital admissions. Diabetic patients undergoing stress from illness or injury may not recognize early ketosis and often present advanced ketoacidosis, requiring intensive care admission. We have recently developed a microneedle-based technology to extract dermal interstitial fluid (ISF) from both animals and humans, which could enable wearable sensors to rapidly detect ketosis. Metabolite concentrations in ISF may differ in urine and blood and could likely represent local metabolic conditions in the surrounding tissue. Development of a wearable ketone detector will require an understanding of ketone concentrations and kinetics in ISF. Here, we report data that is first of its kind, with regard to the ketone concentrations present in the dermal ISF of rats, their correlation to blood, and the possible impact on the development of a wearable ISF “early warning system” to prevent morbidity from DKA. We extracted ISF, using minimally invasive microneedle arrays, from control Sprague Dawley rats and 17 h fasted rats. ISF and blood ketone levels were measured using a common glucose/ketone meter and strips. Local tissue concentrations of glucose were similar to those of blood, with an average blood to ISF glucose ratio of 0.99 ± 0.15 mg/dL. ISF ketones (0.4 ± 0.3 mM) were significantly higher (*p* = 4.2 × 10^−9^), compared with blood ketones (0.0 ± 0.0 mM). Although the fasted animals had slightly higher ISF ketones (1.3 ± 1.1 mM) compared with blood ketones (1.0 ± 1.0 mM), the difference was not significant (*p* = 0.3). This suggests ISF could possibly be useful as a surrogate for blood when determining ketone levels within a clinical setting.

## 1. Introduction

Diabetic ketoacidosis (DKA) is one of the most dangerous and expensive complications of diabetes [1,2,3,4], and a 30% increase in DKA incidence has been documented over the past decade [2]. Earlier diagnosis, treatment, and management of DKA are of vital importance to improve patient outcomes [2]. DKA mortality rates in children with undiagnosed Type-I diabetes are as high as 40% [3,4]. Moreover, 15–20% of adult and 30–40% of adolescent diabetic patients with undiagnosed diabetes, first present with DKA [2]. DKA is characterized by an increase in ketone bodies as cells become unable to transport and use glucose [2,5,6]. In the emergency department (ED), the key laboratory findings of DKA generally include glucose levels greater than 250 mg/dL, blood pH less than 7.3, serum bicarbonate less than 18 mmol/L, anion gap less than 10, and the presence of urine and serum ketones. Blood sampling, for ketones, typically tests for β-hydroxy-butyrate (BHB), which is the predominant ketone species found in blood. Urine sampling, for ketones, tests for acetoacetate, which is the predominant ketone species found in urine. Although urine has been proposed as a potential fluid of interest, it represents average systemic concentrations and can lag real time ketone concentrations in the blood and possibly interstitial fluid. Additionally, subjects suffering from diabetic ketoacidosis are often dehydrated, and thus, alternate means of quantifying ketones in other fluids are needed. Teymourian et al. demonstrated the development and use of a continuous ketone-bodies sensor [7]; however, the device was tested using an artificial ISF and gel phantoms in vitro. To date, we are unaware of any reports on ketone concentrations or kinetics in dermal interstitial fluid (ISF) in vivo and how these compare with blood.

Direct measurement of ketones in the blood requires blood sampling and lab turn-around times of one or more hours. Urine testing is least invasive, but an increase in urine ketone levels may lag increases in blood ketone levels. Additionally, urine testing is not amenable to continuous monitoring. Finally, due to its lowered buffering capacity, ISF can have changes in pH that are greater than, and develop more rapidly than, that of blood [5] (normal blood and ISF pH are 7.35–7.45 and 6.6–7.6, respectively); therefore, ISF pH level changes may happen more rapidly, and be a better, faster diagnostic indicator of potential ketoacidosis than blood or urine [5]. A wearable, minimally invasive, ISF sensor for ketone bodies and/or pH could potentially enable better treatment and management of DKA. We hypothesized that BHB in ISF could serve as a convenient alternative to blood testing and monitoring, while enabling the development of a minimally invasive and wearable early warning system for DKA.

We recently reported on a method to extract ISF from both humans and animals using microneedles. This approach provides adequate volumes of ISF for downstream proteomic, transcriptomic, and extracellular vesicle analyses by employing microneedle array (MA) technology [7,8,9]. The microneedle technology for this study utilized a 5-needle and a 3D-printed MA holder that allows for the application of 5 needles at a time into the dermis to extract ISF. We have also successfully used this technology to extract ISF from human subjects under approved protocols [9,10]. Additionally, we have performed extensive research into the optimal anatomical regions for ISF extraction, as well as our needle performance statistics [8]. Although several different microneedle-extraction techniques for ISF have been previously reported [11,12], damage to tissues due to blistering and/or negative pressure, as well as the small amount of ISF collected, limit the usefulness of these techniques [13,14]. Our novel microneedle extraction technique allows for collection of 10 µL of ISF in under 30 min. Microneedles do not penetrate the dermis deep enough to contact the blood supply or nerves, and thus, are much less painful and invasive than blood sampling [11,15]. Additionally, we have been able to successfully extract 15–20 µL of ISF from humans using our MA technology [9,10]. We now aim to expand this ISF characterization to metabolomics and to better understand the biochemical changes in ISF during ketoacidosis towards the development of a wearable, minimally invasive MA for detecting and monitoring DKA.

## 2. Results

### 2.1. MA Prototype Design and Testing

**Figure 1:**
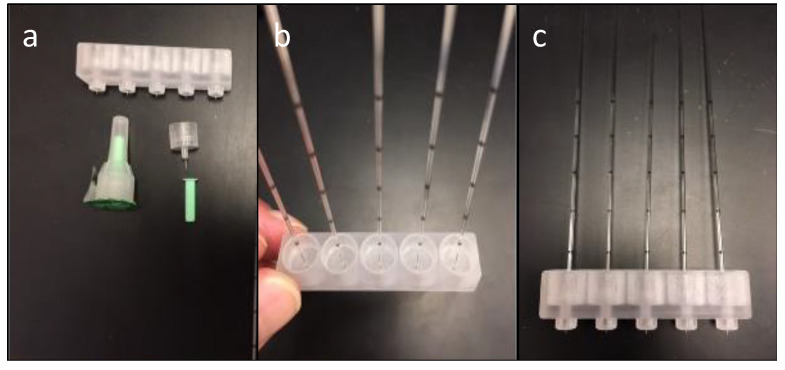
MA Design. Microneedle, capillary, and MA with needles inserted. (**a**) Top (**b**) and side (**c**) views of fully assembled MA.

We first produced MAs by designing and 3D printing linear prototypes. These were tested in animals to produce a working prototype for the experiments [16]. Our MA extraction approach collected 60–100 µL of ISF from single animals over 1–2 h while avoiding blistering or suction. Figure 1 shows our 3D printed microneedle array holders, needle assembly, and application in CD Hairless rats.

### 2.2. In Vivo ISF and Blood Glucose Measurements in Fasted Rats

**Figure 2:**
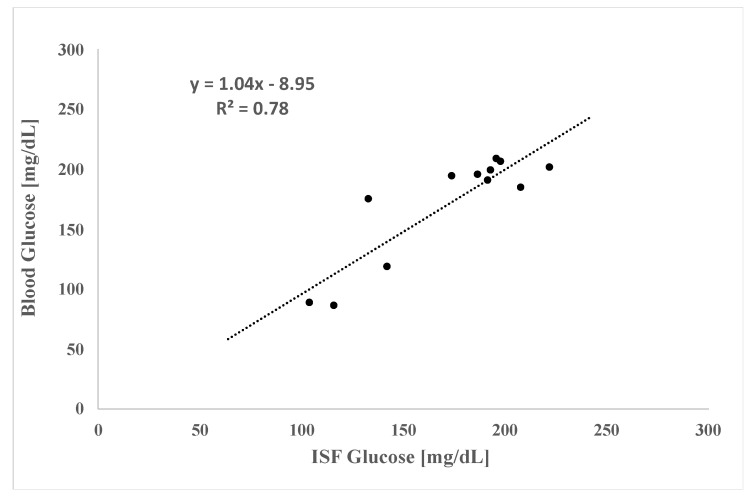
ISF and Blood Glucose Correlations in 17 h Fasted CD Hairless Rats. Number of animals (N) = 2; number of total measurements (n) = 12 for each fluid. Samples were collected over a 1.8 h ISF extraction period while the animals remained under 2% isoflurane anesthesia applied via a nosecone. The dashed line is the linear fit line. The equation and R^2^ value are shown in the top left corner of the plot.

Ketosis is not only a consequence of untreated and/or poorly managed diabetes but is also a consequence of fasting; therefore, to increase blood ketones in the CD Hairless rats, we chose a simple overnight fasting model, to increase the concentration of ketones in the blood and ISF. Two animals fasted for 17 h, and three control animals had not fasted. After the fasting period, animals were anesthetized with isoflurane, and ISF was continually extracted with MAs over a 2 h period while blood and ISF ketones and glucose were measured in real time using a glucose/ketone meter. We first examined the blood glucose and ketone levels over 1.8 h of isoflurane anesthesia. We found that local tissue concentrations of glucose were similar to those of blood (Figure 2), with an average blood to ISF glucose ratio of 0.99 ± 0.15 mg/dL. Linear regression analysis of blood versus ISF glucose levels suggest robust correlation (R^2^ = 0.78). Others have described a lag time for glucose in the tissue (ISF) compared with blood, [17] though we did not attempt to collect kinetic data for glucose levels in this study.

### 2.3. In Vivo Ketone Measurements in Fasted vs. Control Animals

**Figure 3:**
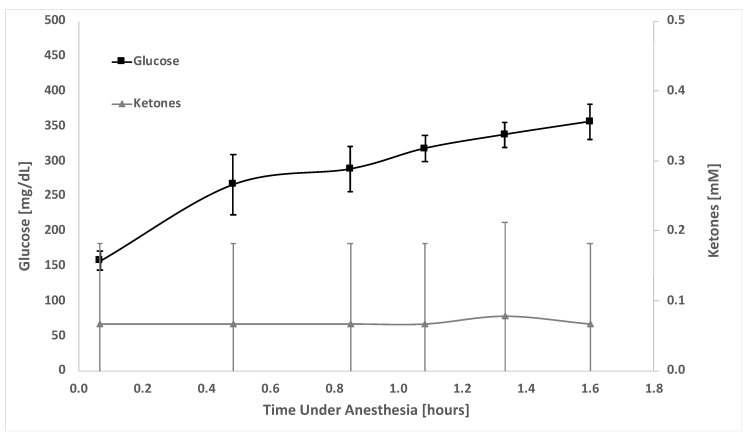
Ketone and glucose levels in the blood of control CD hairless rats. Ketones and glucose were measured in blood (via a tail snip), using a glucose/ketone test meter, at different time intervals over a 2.0 h ISF extraction experiment. Triplicate measurements were made for each metabolite at each time point. Glucose (squares, black); ketones (triangle, grey). Number of animals (N) = 3. Left y-axis shows glucose in mg/dL whereas the right y-axis shows ketones in mM. Time the animal was under 2% isoflurane anesthesia is shown on the x-axis.

Figure 3 shows ketone and glucose measurements for blood in only three control (non-fasted) animals. Measurements are shown for the animals over a 1.6 h period while they remained under 2% isoflurane anesthesia. Others have previously shown that metabolites, such as glucose, can increase in animals exposed to anesthesia [18]. Data in Figure 3 suggest that the ketone concentrations (0.1 ± 0.1 mM) did not fluctuate while the animals were under anesthesia; however, glucose increased from 157 ± 13 mg/dL at the beginning of the procedure to 356 ± 25 mg/dL after 1.6 h of anesthesia, in accordance with prior reports [18].

**Figure 4:**
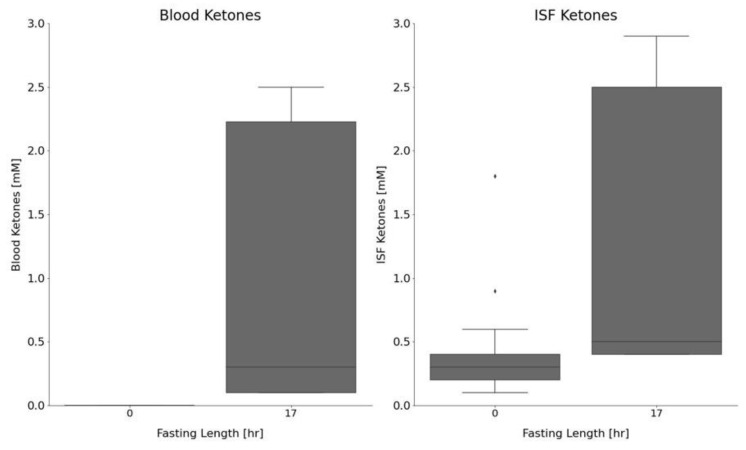
ISF and blood ketones measured in control (non-fasted) and fasted (17 h) CD hairless rats (fasted: n = 2; control: n = 3). Boxplots showing the median (solid line), minimum (bottom whisker), lower quartile (bottom of box), upper quartile (top of box), maximum (upper whisker), outliers (dots), and interquartile range (gray).

We next examined both the blood and ISF ketone and glucose concentrations in fasted versus control animals. Animals fasted for 17 h saw a significant decrease in glucose, compared with the control animals (155 ± 44 mg/dL and 318 ± 86 mg/dL, respectively (*p* = 2.09 × 10^−10^)). Moreover, blood ketones were significantly elevated in the fasted rats, compared with the control animals (1.0 ± 1.0 mM and 0 ± 0 mM, respectively (*p* = 9.6 × 10^−7^)). Likewise, ISF ketones were significantly elevated in the fasted versus control animals (1.3 ± 1.1 mM and 0.4 ± 0.3 mM (*p* = 9.96 × 10^−6^)). ISF and blood ketone measurements are shown in Figure 4 for both the control and 17 h fasted animals.

**Figure 5:**
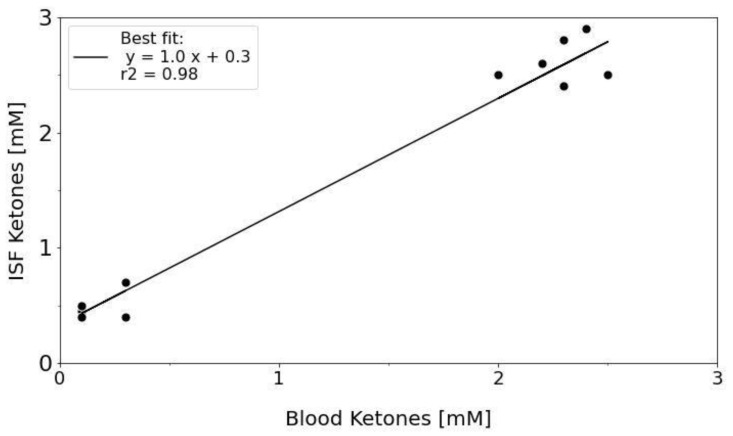
ISF and blood ketone correlation for fasted (17 h) CD hairless rats (N = 2). Dots show the ISF/Blood measurements whereas the black line shows the best linear fit line. The linear fit equation and r^2^ values are shown in the top left corner of the figure.

We also compared the ketone concentrations measured in blood and ISF between the control animals. ISF ketones (0.4 ± 0.3 mM) were significantly higher (*p* = 4.2 × 10^−9^), compared with blood ketones (0.0 ± 0.0 mM). Although the 17 h fasted animals had slightly higher ISF ketones (1.3 ± 1.1 mM), compared with blood ketones (1.0 ± 1.0 mM), the difference was not significant (*p* = 0.3). This suggests that the ISF could possibly be useful as a surrogate for blood in determining ketone levels within a clinical setting. Table 1 lists the average glucose and ketone measurements for the different cohorts of animals. We also compared the correlation between ISF and blood ketone concentrations in the 17 h fasted group of animals (Figure 5). The data suggests a good correlation (r^2^ = 0.98) between the ISF and blood ketones with the ISF ketone concentrations trending upward by 0.3 mM (intercept). The higher levels of ketones found in the ISF versus blood, suggest that an increase in ISF ketones may be detectable before an increase in blood ketones, possibly leading to an earlier diagnosis of ketosis through ISF monitoring. To our knowledge, this is the first report on in vivo ISF ketone measurements and the first time ISF ketone levels have been correlated with blood in vivo.

## 3. Discussion

Our recent advances in ISF extraction and analysis—developed in animal models and tested in healthy human subjects—have established a minimally-invasive method that can be adapted to monitor metabolites. We have developed both a microneedle array to extract ISF and accompanying methodologies for ISF analysis, and we sought to apply this approach to begin to understand the metabolomic signature of ISF. We ultimately envision a wearable microneedle patch that could be used to monitor ketoacidosis recovery or act as an early warning system of increasing ketones in individuals with diabetes.

Although microneedle technology has found potential applications in glucose monitoring and drug delivery, the molecular composition of ISF remains incompletely characterized. Only recently have studies begun reporting on some of the proteomic, transcriptomic, and vesicular components in ISF; however, in vivo metabolic studies of ISF, beyond glucose, are lacking. Our central hypothesis is that MA extraction of ISF will enable minimally invasive quantitation of ketones, which will be a highly stable metric for ketoacidosis severity. We extracted ISF and measured ketones and glucose in the ISF and blood of fasted control rats.

Fasting led to a significant increase in fluid ketones, and this increase was similar between ISF and blood, although ISF ketone levels tended to be higher than blood levels. This suggests that ISF ketones may also be similar to blood ketones in the clinic, and that elevations in ISF ketones could precede elevations in the blood. Our laboratory and others have found that the blood to ISF glucose ratio is approximately 1.0; however, we found in the CD hairless rat strain, that the blood to ISF ketone ratio was 0.73, suggesting higher concentrations in the ISF. Future human studies, as well as further animal studies evaluating ISF ketone concentrations, kinetics, and dynamics, would aid in further elucidating the correlation between ISF and blood ketones. This was an initial feasibility study with a small sample size; however, this will suffice to perform a power calculation for future studies in which test characteristics such as sensitivity, specificity, as well as negative and positive predictive values can be measured. Additionally, future studies to determine whether the device could have dual purposes for microinfusions of insulin with simultaneous ketone quantification are warranted. To our knowledge, this is the first report on ketones that has been measured in unaltered dermal ISF in vivo, and the first time ISF ketone levels have been correlated with blood ketone levels in vivo. Importantly, the slightly higher levels of ketones found in the ISF versus blood, suggest that an increase in ISF ketones may be detectable before an increase in blood ketones, possibly leading to better treatment and management of ketosis through ISF monitoring.

## 4. Materials and Methods

### 4.1. Animals, Materials, and Supplies

The animal care and use program of the University Of New Mexico (UNM) is accredited by AAALAC International and the UNM’s animal care and use committee approved all experiments. Five CD hairless, Crl:CD-Prss8hr, rats (Charles River Laboratories, Wilmington, MA, USA) were used for the studies. In general, rats are used instead of mice because the dermal thickness and histology more closely approximates human skin. Additionally, a larger surface area for microneedle application is required for optimizing extraction methods.

### 4.2. Fasting Model

The animals initially had unlimited access to normal water and a standard rat diet. Fasted rats (n = 2) had food withdrawn for 17 h, whereas control rats (n = 3) continued to have ad lib access to food. At the conclusion of the fasting period, animals were anesthetized and had ISF extracted using the MAs.

### 4.3. MA Design and 3D Printing

Fusion360 and SketchUp computer aided design (CAD) software was used for designing the MAs and designs were exported to an object file (.stl) using 3D Sprint (3D Systems, Inc., Rock Hill, SC, USA). The print resolution is highest in the z direction which is 0.02 mm. MA holders were 3D printed using a commercially available ProJet MJP 2500 printer (3D Systems, Inc.) using a VisiJet^®^ M2R-CLR build material and a VisiJet^®^ M2 SUP support material, both from 3D Systems. After printing, the MA holders were placed at −20 °C for 5 min to release the printed holders from the base plate. MA holders were then placed in a steam bath for 15 min to remove the wax support material and subsequently placed in a hot oil bath for another 15 min to remove all traces of the wax support. MA holders were then cleaned using hot tap water and soap and left at room temperature to dry.

### 4.4. ISF Extraction and Fluid Collection

ISF was extracted using our previously published methods [8,9,10]. Briefly, animals were anesthetized using 2% isoflurane. Microneedle arrays were then applied to extract ISF. Ultra-fine Nano PEN needles (BD, Franklin Lakes, NJ, USA) were placed into 3D-printed microneedle array holders. Each needle was attached to a 1–5 µL calibrated pipet capillary tube (Drummond Scientific Co., Broomall, PA, USA). The array assembly was then pressed into the dermal tissue of the rats and held in place until enough ISF was collected. ISF was transferred from the capillary tubes onto the glucose or ketone meter strips using a small plunger that is supplied with the capillary tubing. Glucose and ketone measurements were immediately recorded using a Nova Max Plus glucose/ketone meter (Nova Biomedical, Waltham, MA, USA) with Nova Max Plus glucose and ketone test strips (Nova Biomedical, Waltham, MA, USA), and another ISF extraction was then performed. This routine was repeated for 1.8 h for multiple glucose and ketone measurements of both blood and ISF. The blood was obtained from a small tail snip, where a drop of blood from the tail was periodically placed on the glucose and ketone test strips to measure the blood concentrations correlating with the times of ISF measurement. All animals had a terminal cardiac puncture performed at the conclusion of each experiment.

### 4.5. Data Analysis

All analyses were performed in either Microsoft Excel or Python. Pandas, Numpy, Matplotlib.pyplot, Seaborn, and Scipy libraries were utilized within Python. All *p*-values were calculated using 2-tailed T-tests with equal variance.

## Figures and Tables

**Table 1:** Average glucose and ketone measurements for fasted versus non-fasted rats.

	Control	17 h Fast
**Number of animals (N)**	3	2
**Glucose**		
**Number of samples (n)**	34	20
**Mean Glucose [mg/dL]**	318	155
**Stdev Glucose [mg/dL]**	86	44
**Blood Ketones**		
**Number of samples (n)**	34	20
**Mean Blood Ketones [mM]**	0.0	1.0
**Stdev Blood Ketones [mM]**	0.0	1.0
**ISF Ketones**		
**Number of samples (n)**	31	17
**Mean ISF Ketones [mM]**	0.4	1.3
**Stdev ISF Ketones [mM]**	0.3	1.1

## Data Availability

The data presented in this study are available on request from the corresponding author due to restrictions on privacy.
